# Supplementary material for: Identification of triple-negative breast cancer cell lines classified under the same molecular subtype using different molecular characterization techniques: Implications for translational research
Source: PLoS One. 2020 Apr 30;15(4):e0231953. doi: 10.1371/journal.pone.0231953 (PMC7192374; doi:10.1371/journal.pone.0231953)
Supplement: S1 Table — (DOCX) [file pone.0231953.s001.docx]

**Supplementary Table 1. Correlation values from each subtype of the 6 molecularly stable TNBC cell lines and xenografts derived from multiple sources according to classification with the TNBCtype-IM algorithm**

| **Cell Line** | **Source** | **BL1_corr** | **BL2_corr** | **LAR_corr** | **M_corr** | **MSL_corr** | **IM_corr** |
| --- | --- | --- | --- | --- | --- | --- | --- |
| HCC70 | Xenograft | 0.02 | 0.38 | -0.03 | 0.01 | -0.42 | -0.09 |
| HCC70 | GSE15361 | 0.22 | 0.23 | -0.42 | 0.13 | -0.19 | 0.04 |
| HCC70 | CCLE | 0.25 | 0.27 | -0.18 | -0.14 | -0.41 | 0.13 |
| SUM149PT | Xenograft | -0.11 | 0.37 | -0.10 | 0.19 | -0.30 | -0.22 |
| SUM149PT | GSE15361 | 0.06 | 0.14 | -0.25 | 0.14 | -0.22 | 0.00 |
| SUM149PT | CCLE | 0.00 | 0.21 | -0.13 | 0.17 | -0.28 | -0.14 |
| HCC1806 | Xenograft | -0.22 | 0.49 | -0.04 | 0.23 | -0.25 | -0.24 |
| HCC1806 | GSE15361 | NA | NA | NA | NA | NA | NA |
| HCC1806 | CCLE | 0.07 | 0.26 | -0.14 | -0.05 | -0.27 | 0.10 |
| BT549 | Xenograft | -0.11 | 0.08 | -0.19 | 0.41 | 0.16 | -0.33 |
| BT549 | GSE15361 | 0.05 | 0.08 | -0.22 | 0.18 | 0.08 | 0.05 |
| BT549 | CCLE | 0.02 | 0.03 | -0.05 | 0.15 | -0.11 | -0.05 |
| MDAMB453 | Xenograft | -0.13 | 0.08 | 0.38 | -0.20 | -0.30 | -0.08 |
| MDAMB453 | GSE15361 | -0.17 | -0.19 | 0.30 | -0.15 | 0.13 | 0.03 |
| MDAMB453 | CCLE | -0.05 | -0.15 | 0.40 | -0.17 | -0.21 | -0.03 |
| HCC2157 | Xenograft | 0.33 | -0.14 | -0.22 | 0.29 | -0.51 | -0.21 |
| HCC2157 | GSE15361 | 0.44 | -0.31 | -0.31 | 0.21 | -0.25 | 0.02 |
| HCC2157 | CCLE | 0.40 | -0.23 | -0.19 | 0.13 | -0.39 | -0.01 |
