# Supplementary material for: Identification of triple-negative breast cancer cell lines classified under the same molecular subtype using different molecular characterization techniques: Implications for translational research
Source: PLoS One. 2020 Apr 30;15(4):e0231953. doi: 10.1371/journal.pone.0231953 (PMC7192374; doi:10.1371/journal.pone.0231953)
Supplement: Attachment — Submitted filename: Response to Reviewers PONE-D-19-21744.pdf [file pone.0231953.s002.pdf]

THE UNIVERSITY OF TEXAS
MD Anderson Cancer Center
Making Cancer History®

Naoto Ueno, MD, PhD, FACP
Professor, Breast Medical Oncology
Nylene Eckles Distinguished Professor in Breast Cancer Research
Chief, Section of Translational Breast Cancer Research
Executive Director of the Morgan Welch Inflammatory Breast Cancer Research Program

nueno@mdanderson.org
D 713-792-8754 C 713-401-9238

Department of Breast Medical Oncology
T 713-792-2817 F 713-794-4385
Unit 1354
1515 Holcombe Boulevard
Houston, Texas 77030

December 12, 2019

Dr. Joerg Heber
Editor-in-Chief
PLOS ONE Editorial Office
1160 Battery Street
Koshland Building East, Suite 225
San Francisco, CA 94111
United States
Phone: +1 (415) 624-1200 | Fax: +1 (415) 546-4090

Dear Dr. Heber,

Thank you for the opportunity to revise our manuscript *"Identification of triple*-*negative breast cancer cell lines classified under the same molecular subtype using different molecular characterization techniques: implications for translational research."* We have addressed the academic editor's and reviewers' concerns, as indicated by our point-by-point responses to the comments in italics below.

Journal Requirements:

1. When submitting your revision, we need you to address these additional requirements.
Please ensure that your manuscript meets PLOS ONE's style requirements, including those for file naming. The PLOS ONE style templates can be found at
http://www.journals.plos.org/plosone/s/file?id=wjVg/PLOSOne_formatting_sample_main_body.pdf
and
http://www.journals.plos.org/plosone/s/file?id=ba62/PLOSOne_formatting_sample_title_authors_affiliations.pdf

We have renamed files and addressed the manuscript style to meet PLOS ONE's requirements.

2. At this time, we request that you please report additional details in your Methods section regarding animal care, as per our editorial guidelines: 1) Please provide details of animal welfare (e.g., shelter, food, water, environmental enrichment) 2) please describe any steps taken to minimize animal suffering and distress, such as by administering anaesthetics or analgesics, 3) please include the method of sacrifice and 4) Please describe the frequency of monitoring and the criteria used to assess animal health and well-being. Thank you for your attention to these requests.

We have added the following to our methods section:

Human xenograft tumors were established in 4-6 week old immunocompromised mice (Nod-SCID-Gamma) that were bred in-house (Department of Experimental Radiation Oncology, MD Anderson Cancer Center) and housed in pathogen-free conditions within the MD Anderson Research Animal Support Facility. Mice were treated in accordance with NIH guidelines and received standard chow and water ad libitum. Individual tumor xenografts were established in anesthetized mice by implanting 5 × 106 TNBC cells, re-suspended in a 50:50 Matrigel:PBS solution, into the fourth inguinal mammary gland. Established tumors were monitored three times weekly by caliper measurements, and mice euthanized by CO2 asphyxiation when tumors reached 750 mm3. (lines 113 to 123)

3. We note you have included a table to which you do not refer in the text of your manuscript. Please ensure that you refer to Table 1 in your text; if accepted, production will need this reference to link the reader to the Table.

Thank you for the suggestion. We have referred to Table 1 in the Introduction section, line 72.

4. Thank you for stating the following in the Competing Interests section:
I have read the Journal's Policy and the author of this manuscript have the following competing interests: NTU declares contracted research with Insight Genetics, Inc.. RSS, DRH, BLS, TJN, ORL are all employee of Insight Genetics. The other authors declare no potential conflicts of interest.
We note that one or more of the authors are employed by a commercial company: Insight Genetics, Inc.
1. Please provide an amended Funding Statement declaring this commercial affiliation, as well as a statement regarding the Role of Funders in your study. If the funding organization did not play a role in the study design, data collection and analysis, decision to publish, or preparation of the manuscript and only provided financial support in the form of authors' salaries and/or research materials, please review your statements relating to the author contributions, and ensure you have specifically and accurately indicated the role(s) that these authors had in your study. You can update author roles in the Author Contributions section of the online submission form.
Please also include the following statement within your amended Funding Statement.
"The funder provided support in the form of salaries for authors [insert relevant initials], but did not have any additional role in the study design, data collection and analysis, decision to publish, or preparation of the manuscript. The specific roles of these authors are articulated in the 'author contributions' section."
If your commercial affiliation did play a role in your study, please state and explain this role within your updated Funding Statement.
2. Please also provide an updated Competing Interests Statement declaring this commercial affiliation along with any other relevant declarations relating to employment, consultancy, patents, products in development, or marketed products, etc.
Within your Competing Interests Statement, please confirm that this commercial affiliation does not alter your adherence to all PLOS ONE policies on sharing data and materials by including the following statement: "This does not alter our adherence to PLOS ONE policies on sharing data and materials." (as detailed online in our guide for authors http://journals.plos.org/plosone/s/competing-interests) . If this adherence statement is not accurate

and there are restrictions on sharing of data and/or materials, please state these. Please note that we cannot proceed with consideration of your article until this information has been declared. Please include both an updated Funding Statement and Competing Interests Statement in your cover letter. We will change the online submission form on your behalf.
Please know it is PLOS ONE policy for corresponding authors to declare, on behalf of all authors, all potential competing interests for the purposes of transparency. PLOS defines a competing interest as anything that interferes with, or could reasonably be perceived as interfering with, the full and objective presentation, peer review, editorial decision-making, or publication of research or non-research articles submitted to one of the journals. Competing interests can be financial or non-financial, professional, or personal. Competing interests can arise in relationship to an organization or another person. Please follow this link to our website for more details on competing interests: http://journals.plos.org/plosone/s/competing-interests

To address PLOS ONE's requirements, we have edited our Funding Statement and Competing Interests Statement as follows:

Funding Statement: This study was supported by the Morgan Welch Inflammatory Breast Cancer Research Program, a State of Texas Rare and Aggressive Breast Cancer Research Program Grant, and National Cancer Institute award P30CA016672, which supports the MD Anderson Cancer Center Clinical Trials Support Resource. NTU has contracted research with Insight Genetics Inc. RSS, DRH, BLS, TJN, ORL are all employees of Insight Genetics, Inc. All funders in this study provided support in the form of research materials, but did not have any additional role in the study design, data collection and analysis, decision to publish, or preparation of the manuscript. The specific roles of these authors are articulated in the 'author contributions' section

Competing Interests Statement: Naoto T. Ueno declares contracted research with Insight Genetics, Inc. RSS, DRH, BLS, TJN, ORL are all employee of Insight Genetics, Inc. This affiliation does not alter our adherence to PLOS ONE policies on sharing data and materials. Insight Genetics Inc. provided support in the form of research materials, but did not have any additional role in the study design, data collection and analysis, decision to publish, or preparation of the manuscript. The specific roles of these authors are articulated in the 'author contributions' section. All other authors declare no potential conflicts of interest.

Reviewers' comments:

Reviewer #1: PONE-D-19-21744
The manuscript by Fernandez and colleagues relays important information for the molecular classification of TNBC subtypes in cell line models. There are a number of points that should be clarified and/or expanded prior to publication.

1. The methods indicate that publicly available gene expression data were used to classify the 28 TNBC cell lines. The authors should explicitly state which publically available data. Two references

were cited. How was the data from the different articles analyzed? Analysis of this data should be more thoroughly explained in the methods section. Additionally, could the use of publicly available data have an impact on discordant results between cell line and xenograft results? For discordant results did the authors repeat RNA sequencing from cell lines grown in their own laboratory and compare to their RNA sequencing analysis from the xenograft studies presented here?

We appreciate the reviewer's comment. Normalized data from the CCLE (RNAseq), GSE-10890, and E-TABM-157 publicly available gene data sets were used to classify the 17 TNBC cell lines using both the original 2,188-gene algorithm (TNBCtype) and the 101-gene TNBCtype-IM algorithm. We added a reference for this data sets: Neve RM, Chin K, Fridlyand J, Yeh J, Baehner FL, Fevr T, et al. A collection of breast cancer cell lines for the study of functionally distinct cancer subtypes Cancer Cell. 2006; 10(6): 515–527, where the methods are explained.

In terms of discordance, it was our intention to find TNBC cell lines that would remain stable with regard to molecular subtype regardless of the algorithm or type of model (*in vitro, in vivo*) used. Please also see our responses below to reviewer 2.

2. Figure 1: could the authors please indicate which cell lines are represented in Figure 1. Also, please include the data for each cell line in a supplemental file.

To avoid confusion, we decided to eliminate Figure 1from the manuscript because it does not represent any particular cell line data. Also, the correlation values for each cell line have been added to Table 2.

3. In Table 1 it would be helpful to annotate which cell lines were classified as dual subtypes.

We thank the reviewer for this comment. No cell line data results are described in Table 1. However, we have annotated dual subtypes in Table 2 and added correlation values.

4. The authors should present the analysis using TNBCtype from the xenograft data in addition to the TNBCtype-IM (Table 3).

We agree with the reviewer. We have added the results of TNBCtype analysis from the xenograft data to table 2 to further demonstrate molecular subtype stability.

5. Page 9, line 177 indicates that 6 of 17 tumor xenograft models tested were concordant, but in Table 3 HCC70 cell line and xenograft data also appears to be concordant for BL2.

We agree with the reviewer and have updated our discussion of the results in Table 2 accordingly.

6. Please clarify, were xenografts only established from 17/28 because 11 of the cell lines did not grow in vivo? Also clarify, was xenograft tumor data from an n=1 for each cell line?

Only 17 xenografts were established because those are the animal models we had available in our research laboratory. To reduce confusion, we have eliminated all mentions of the 11 cell lines that were not used for xenograft tumor generation. The data from these cell lines (formerly in Table 2) are largely redundant with the xenograft table and data did not meet the bar set forth by this manuscript of demonstrating subtype stability. Various changes have been made in the manuscript as a result of our decision to not mention the 11 cell lines for which xenografts were not established.

Reviewer #2: Identification of triple negative breast cancer cell lines classified under the same molecular subtype using different molecular characterization techniques: implications of translational research.

Fernandez et al.

Manuscript: PONE-D-19-21744

The study by Fernandez et al. investigates the classification of triple negative breast cancer cell lines grown in vitro and as orthotopic xenograft transplant models in vivo. The goal is to define the TNBC subtypes of these cell lines using the original TNBCtype and modified TNBCtype –IM algorithms. The impact of the proposed studies lies in defining the molecular subtypes in order to provide the scientific community a framework by which to select these lines, either in vitro or in vivo, to model TNBC subtypes for mechanistic and/or preclinical studies. While the current study does address this goal, I have several moderate and minor concerns that I have outlined below.

Major Concerns:
1. The classification strategy is not well described. The authors have not defined the correlation cut-off used to define the subgroup classification (line 154-157) nor have they described the rationale for the selection of that value. Likewise, the authors state (line 153-154): "If more than one subtype surpassed the cut-off value with no significant difference between the two..." It was not clear how the authors determined whether there was a significant difference. In general, the methods should be more clearly written.

We appreciate these comments. To address them, we have added the following to our methods section:

Data was log base 2 transformed (either from RPKM or Affy), followed by centering of each gene across the batch that was being analyzed. Correlation values were then determined by using Spearman's rank order method against a centroid value set for each subtype. The cutoff for each subtype was 0.1 (for consistency between TNBCtype and TNBCtype-IM), all model coefficients and cutoffs were determined using the 14 discovery data sets, as in the original Lehmann et al. analysis [2], and were not altered afterwards [4]. If multiple subtypes exceed a correlation value of 0.1, the z-score method was performed for significance between the subtypes above the cutoff. If there was not a significant difference, then multiple subtypes were reported ranked by their level of correlation. (lines 154 to 163)

To better compare TNBCtype and TNBCtype-IM, we applied the correlation cutoff of 0.1 to TNBCtype-IM to match TNBCtype. This resulted in two xenograft results changing. Please see Table 2.

2. The authors should provide a table of correlation coefficients for each cell lines and each subgroup. While the authors have made the calls for each cell line, it would allow the reader to gauge the strength of the correlation if these data were provided.

We agree with the reviewer and have added these details to Table 2.

3. It is not clear how reproducible these classification calls are. The authors should use an independent dataset (i.e. CCLE RNAseq data) to examine subtype classification for these cell lines. This would provide additional confidence that subtype calls are not dependent on culturing methods, specific growth conditions or technical or experimental variables. This is particularly relevant as subtype calls for 8/28 or 28.6% of the cell lines analyzed in the current study and the original Lehman JCI paper are not concordant using the TNBCtype calls (Table 2 of this study vs. Table 3 in Lehman 2011 JCI paper).

We agree with the reviewer. We have re-analyzed all cell line data using the CCLE RNAseq data have generated the *in vitro* TNBCtype-IM subtypes (updated Table 2). The xenograft samples were analyzed using RNAseq, so this is a more appropriate comparison. In addition, we were not able to use the E-TABM-157 dataset used by Lehman due to several missing genes from the modified 101-gene algorithm (TNBCtype-IM). However, owing to availability, we used GSE-10890 to determine the subtype call for HCC3153 (see lines 130 to 135 and lines 181 to 195)

We do expect discordant calls between the two algorithms for the following reasons:
1. As shown in the Ring paper, using a large dataset, we expect approximately 87% concordance between the two algorithms.
2. TNBCtype-IM allows for a dual subtype call with the IM subtype removed and is included as a modifier for each subtype.
3. Using data previously reported, with histopathological quantification and laser-capture microdissection, it was determined that the IM and MSL subtypes as reported in the original TNBCtype algorithm were likely due to tumor-infiltrating lymphocytes and tumor-associated stromal cells, respectively. Therefore, we are more confident in the calls of TNBCtype-IM, which has been modified and does not bias cohorts towards finding samples of these two subtypes.

Minor Concerns:
1. Figure 1 is not very clear. It also appears to be lacking a legend as well as y-axis labels.

We agree with this comment. We have eliminated this figure and added details to the Methods section.

2. Table 2 and 3 seem somewhat redundant and the data could be merged into a single table.

We agree with the reviewer and have made this modification. See lines 34 to 42.

3. Reference 2 appear to be merged with Reference 1 in the References Cited section.

Thank you for the comment. We have corrected this error.

Reviewer #3: A) Spearman's correlation test was used to determine sub-type categories. A score of .195 was presented in Figure 1 as the cutoff selected; however, that score is considered "weak correlation" at best. Could the author address this as well as why Spearman's was selected over other tests available?

The correlation value cutoffs selected have been determined empirically. We have observed gene expression profiles to change in a monotonic but not necessarily linear relationship between the various subtypes, which is often best correlated using Spearman's test as compared to Pearson's test. We have added this information to the Methods section. (lines 150 to 153)

B) The number of available samples in each subcategory seems rather now. In MSL category only two of the available four was correctly selected.

Using data previously reported, with histopathological quantification and laser-capture microdissection, it was determined that the IM and MSL subtypes as reported in the original TNBCtype algorithm were likely due to infiltrating lymphocytes and tumor-associated stromal cells, respectively. It is because of these data, in combination with the new centering method, that it is possible the MSL subtype was overrepresented with the original TNBCtype algorithm and is more accurately represented using TNBCtype-IM.

Sincerely,

Naoto T. Ueno, M.D., Ph.D., F.A.C.P.
Professor of Medicine
