# Supplementary material for: Identification of triple-negative breast cancer cell lines classified under the same molecular subtype using different molecular characterization techniques: Implications for translational research
Source: PLoS One. 2020 Apr 30;15(4):e0231953. doi: 10.1371/journal.pone.0231953 (PMC7192374; doi:10.1371/journal.pone.0231953)
Supplement: Attachment — Submitted filename: Response to reviewers.pdf [file pone.0231953.s003.pdf]

THE UNIVERSITY OF TEXAS
MD Anderson ~~Cancer~~ Center
Making Cancer History®

Naoto Ueno, MD, PhD, FACP
Professor, Breast Medical Oncology
Nylene Eckles Distinguished Professor in Breast Cancer Research
Chief, Section of Translational Breast Cancer Research
Executive Director of the Morgan Welch Inflammatory Breast Cancer Research Program

nueno@mdanderson.org
D 713-792-8754 C 713-401-9238

Department of Breast Medical Oncology
T 713-792-2817 F 713-794-4385
Unit 1354
1515 Holcombe Boulevard
Houston, Texas 77030

March 5 2020

Dr. Joerg Heber
Editor-in-Chief
PLOS ONE Editorial Office
1160 Battery Street
Koshland Building East, Suite 225
San Francisco, CA 94111
United States
Phone: +1 (415) 624-1200 | Fax: +1 (415) 546-4090

Dear Dr. Heber,

Thank you for the opportunity to revise our manuscript *"Identification of triple-negative breast cancer cell lines classified under the same molecular subtype using different molecular characterization techniques: implications for translational research."* We have addressed the reviewers' concerns, as indicated by our point-by-point responses to the comments in italics below.

Reviewers' comments:

Reviewer #2: The revised manuscript is improved; however a few concerns remain. First, the level of rigor in the subtype calls is not clear. The authors should report the correlation coefficient values for each cell line or xenograft and each TNBC subtype. Given that most of the reported correlation coefficients are low, this would afford the reader additional insight into the strength of the calls and potential concerns with selecting any given model system for subsequent studies. Secondly, while the authors do demonstrate concordance in subtype calls when specific cell lines are grown in vitro or as an in vivo xenograft, it is unclear how reproducible the subtypes call is between multiple datasets. As I noted in my previous review, and in this review, the subtype correlation coffecicients are relatively weak. As such, the investigators should demonstrate that the same cell line, grown in the same way (i.e. in vitro or in vivo) is consistently classified to the same subtype; there are multiple publicly available RNAseq datasets that can be used to complete these studies. If this is not reproducible, there are concerns with the selection of these lines for future studies as growth conditions will undoubtedly vary between laboratories. Finally, the manuscript is relatively well written, but there are a number of awkwardly worded sections and the discussion is rather brief.

We thank the reviewer for the suggestions. First, we are aware that the correlation coefficient values we are reporting are low so in order to offer the reader additional insight into the strength of each subtype call, we have added a supplementary table where we report correlation values from each

subtype of the 6 molecularly stable TNBC cell lines and xenografts derived from multiple sources according to classification with the TNBCtype-IM algorithm.

Secondly, to demonstrate the strength and reproducibility of our results despite the low correlation coefficient values, we reproduced the calls for the six specific concordant cell lines (SUM149PT, HCC1806, SUM149PT, BT549, MDA-MB-453, and HCC2157) using 5 different datasets (GSE15361, GSE10890, CCLE, Xenograft model, and Lehman, 2011). We found that all cell lines were consistently classified to the same subtype across all datasets. We have included this data in the results and discussion section, and we have also added the results and source of each dataset in table 3.

Sincerely,

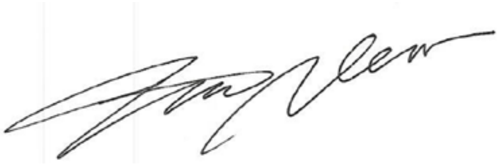

Naoto T. Ueno, M.D., Ph.D., F.A.C.P.
Professor of Medicine
